# Metagenome-assembled genomes of three *Hepatoplasmataceae* provide insights into isopod-mollicute symbiosis

**DOI:** 10.1099/acmi.0.000592.v3

**Published:** 2024-02-20

**Authors:** Satoshi Kawato, Reiko Nozaki, Hidehiro Kondo, Ikuo Hirono

**Affiliations:** ^1^​ Laboratory of Genome Science, Tokyo University of Marine Science and Technology, Tokyo, Japan

**Keywords:** isopods, mollicutes, *Mycoplasma*, symbiosis, metagenome, *Hepatoplasma*

## Abstract

The digestive organs of terrestrial isopods harbour bacteria of the recently proposed mollicute family *Hepatoplasmataceae*. The only complete genome available so far for *Hepatoplasmataceae* is that of ‘*Candidatus* Hepatoplasma crinochetorum’. The scarcity of genome sequences has hampered our understanding of the symbiotic relationship between isopods and mollicutes. Here, we present four complete metagenome-assembled genomes (MAGs) of uncultured *Hepatoplasmataceae* members identified from shotgun sequencing data of isopods. We propose genomospecies names for three MAGs that show substantial sequence divergence from any previously known *Hepatoplamsataceae* members: ‘*Candidatus* Tyloplasma litorale’ identified from the semiterrestrial isopod *Tylos granuliferus*, ‘*Candidatus* Hepatoplasma vulgare’ identified from the common pill bug *Armadillidium vulgare*, and ‘*Candidatus* Hepatoplasma scabrum’ identified from the common rough woodlouse *Porcellio scaber*. Phylogenomic analysis of 155 mollicutes confirmed that *Hepatoplasmataceae* is a sister clade of *Metamycoplasmataceae* in the order *Mycoplasmoidales*. The 16S ribosomal RNA gene sequences and phylogenomic analysis showed that ‘*Candidatus* Tyloplasma litorale’ and other semiterrestrial isopod-associated mollicutes represent the placeholder genus ‘g_Bg2’ in the r214 release of the Genome Taxonomy Database, warranting their assignment to a novel genus. Our analysis also revealed that *Hepatoplasmataceae* lack major metabolic pathways but has a likely intact type IIA CRISPR-Cas9 machinery. Although the localization of the *Hepatoplasmatacae* members have not been verified microscopically in this study, these genomic characteristics are compatible with the idea that these mollicutes have an ectosymbiotic lifestyle with high nutritional dependence on their host, as has been demonstrated for other members of the family. We could not find evidence that *Hepatoplasmataceae* encode polysaccharide-degrading enzymes that aid host digestion. If they are to provide nutritional benefits, it may be through extra-copy nucleases, peptidases, and a patatin-like lipase. Exploration of potential host-symbiont interaction-associated genes revealed large, repetitive open reading frames harbouring beta-sandwich domains, possibly involved with host cell adhesion. Overall, genomic analyses suggest that isopod-mollicute symbiosis is not characterized by carbohydrate degradation, and we speculate on their potential role as defensive symbionts through spatial competition with pathogens to prevent infection.

## Data Summary

The whole genome shotgun sequencing data of isopods are available in DDBJ/ENA/NCBI under the following accession numbers: *Tylos granuliferus*: DRR394944, DRR394945; *Armadillidium vulgare*: DRR394921, DRR394929; *Porcellio scaber*: DRR394922, DRR394930. The complete MAGs of the uncultured mollicutes analysed in this study are available in DDBJ/ENA/NCBI under the following accession numbers: *Candidatus* Tyloplasma litorale Fukuoka2020: AP027078.1; *Candidatus* Hepatoplasma vulgare Av-JP: AP027131.1; *Candidatus* Hepatoplasma scabrum Ps-JP: AP027133.1; *Candidatus* Hepatoplasma crinochetorum Tokyo2021: AP027132.1. Fig. S1, available in the online version of this article, the phylogenetic trees and associated multiple sequence alignments, the ColabFold 3D predictions of selected proteins, and the bioinformatic codes used in this study are available on FigShare https://doi.org/10.6084/m9.figshare.22146599.v4). Fig. S1 and Supplementary Tables are available as Supplementary Material 1.

**Impact Statement**

Terrestrial isopods, commonly known as pill bugs and woodlice, are a unique group of crustaceans that successfully colonized land. Their digestive organs are home to symbiotic microbes that may support the host’s survival. One of the most characteristic microbes associated with terrestrial isopods are members of *Hepatoplasmataceae*, a lineage of mycoplasma-like bacteria (Class *Mollicutes*) that reside on the surface of the host’s midgut gland. It has been suggested that *Hepatoplasmataceae* mollicutes enhance the host’s fitness, but their exact roles remain unknown. Our aim was to better understand their physiological roles by analysing the metagenome-assembled genomes (MAGs) of novel *Hepatoplasmataceae* lineages. We explored the *Hepatoplasmataceae* MAGs using various homology searches to identify enzymes that possibly provide nutritional benefits to the host. This search identified several peptidases, a phosphotrehalase, and a patatin-like lipase, but no polysaccharide-degrading enzymes, contrary to their suspected role in aiding polysaccharide degradation. This raises the possibility that the symbiotic relationship between isopods and *Hepatoplasmataceae* is not primarily defined by the exchange of essential nutrients, as is often the case in insect-bacterial symbiosis. Rather, we suggest that hepatoplasmas are defensive symbionts that limit the growth of pathogenic microbes by occupying the host digestive organs.

## Introduction

Terrestrial isopods (Isopoda: suborder Oniscidea), commonly called woodlice or pill bugs, are a group of crustaceans that have adapted to life on land [1, 2], where they play an important ecological role as decomposers [3]. Not only are they considered as a prime example of evolutionary transitions from aquatic and terrestrial lifestyles, but they have served as model organisms to study sex determination [4–7], mitochondrial genome architecture [8–10], and symbiosis [11–14].

The digestive organs of terrestrial isopods are home to various symbiotic microorganisms that are thought to enhance the host’s fitness [11, 12, 14–17]. These symbionts have drawn researchers’ attention mainly due to their possible roles in aiding digestion and nutrition, as dead plant material, their main feed, are considered to be nutritionally poor.

*Candidatus* Hepatoplasma [18] (*Mollicutes: Hepatoplasmataceae* [19]) are one of the most well-characterized isopod symbionts, which reside on the brush borders of the host’s hepatopancreas (also called midgut caeca) [15]. There is some evidence that hepatoplasmas are mutualistic symbionts of isopods, as they are found in a variety of terrestrial and semiterrestrial isopods and have the signature of host-symbiont co-evolution [12]. Additionally, the presence of hepatoplasmas is correlated with a higher survival rate under a low-quality diet [12]; this has led to speculation that hepatoplasmas are nutritional symbionts that provide nutritional benefit to the host. However, the exact physiological advantage of harbouring these mollicutes remains unclear.

Limited genome data is available for hepatoplasmas, which has hampered our understanding of the symbiotic relationship between isopods and mollicutes. The only complete genome available for *Hepatoplasmataceae* available to date is that of ‘*Candidatus* Hepatoplasma crinochetorum’ [18], in which the authors analysed its phylogenetic position within *Mollicutes*, peculiar organization of tryptophan transfer RNA (tRNA) genes, and probable lack of CRISPR/Cas system. Several draft metagenome-assembled genomes of *Hepatoplasmataceae* members have been reported [19–21]. Collingro *et al*. [20] reported on the draft genome sequence of *Candidatus* Hepatoplasma crinochetorum isolate Ps and noted the presence of a type I restriction modification system and the lack of CRISPR/Cas system. Wang *et al*. [21] reported on two *Hepatoplasmataceae* MAGs identified from the stomach of deep-sea isopod *Bathynomus* sp. and suggested that they may be helping the host survive in low-nutrient conditions, based on the presence multiple copies of genes related to proteolysis and oligosaccharide degradation. Aubé *et al*. [19] reported on five *Hepatoplasmataceae* MAGs identified from the foregut of a deep-sea shrimp *Rimicaris exoculata*, noting the highly streamlined genome architectures lacking major metabolic pathways, which suggest that they are a secondary user of complex molecules that have already been broken down.

We hypothesized that additional *Hepatoplasmataceae* genomes would help to understand the genetic basis of the physiological benefits they provide, if any. Here, we present complete metagenome-assembled genomes (MAGs) of four *Hepatoplasmataceae* representatives, three of which are potentially novel species. Genomic analysis supports the view that hepatoplasmas are ectosymbionts with high nutritional dependence on the host. *Hepatoplasmataceae* MAGs lack polysaccharide-degrading enzymes, and if they are to provide nutritional benefits to the host, it would be through several nucleases, peptidases, and a lipase, which are of unknown functions. Given the lack of definitive evidence that hepatoplasmas provide nutritional benefits to the host, we suggest that hepatoplasmas are more likely to be defensive symbionts, which compete and limit the growth of other pathogenic microorganisms.

## Methods

### Isopod origin and genome sequencing

Twenty *Tylos granuliferus* animals, originating from Fukuoka Prefecture, Japan, were purchased from an amateur collector in October 2020. Ten each of *Armadillidium vulgare* and *Porcellio scaber* animals were caught at the Shinagawa Campus, Tokyo University of Marine Science and Technology, Japan, in 2021. For all three isopod species, the animals were starved in a humidified chamber for several days before DNA extraction. Total (i.e. holobiont) DNA was extracted from a single animal per species by standard phenol-chloroform-isoamyl alcohol extraction (*T. granuliferus*) [22] and MagAttract HMW DNA Kit (Qiagen) (*A. vulgare* and *P. scaber*). Nanopore sequencing libraries were prepared using the Ligation Sequencing Kit (SQK-LSK109) according to the manufacturer’s instructions and were sequenced on R9.4.1 flow cells. The ONT .fast5 files were base-called using Guppy v. 5.0.13 in super accuracy mode. The same DNA preparations were sequenced on a HiSeq 4000 instrument (2×150 bp paired-end) by Eurofins Genomics (Tokyo, Japan). The Illumina reads were quality filtered using Fastp v. 0.21.0 prior to use [23].

### General assembly and annotation strategy

The ONT and Illumina reads were separately assembled and screened for possible symbiont genomes. Three MAGs (*Candidatus* Tyloplasma litorale Fukuoka2020, *Candidatus* Hepatoplasma vulgare. Av-JP, and *Candidatus* Hepatoplasma crinochetorum Tokyo2021) were extracted from ONT assemblies and polished using Illumina reads. The MAG of *Candidatus* Hepatoplasma scabrum Ps-JP was extracted from an Illumina assembly as a circular contig, which was not subjected to further polishing. Recovered as circular contigs, these MAGs were considered complete genomes and individually annotated like typical isolate genomes.

### Genome assembly of ‘*Candidatus* Tyloplasma litorale Fukuoka2020’

The *T. granuliferus* ONT reads were filtered using SeqKit [24] at lengths of 5, 10, and 20 kb, and the three sets of length-filtered reads were *de novo* assembled by metaFlye v. 2.8.3 [25]. The three assemblies all contained a circular, mollicute-like contig with a length of approximately 600 kb. The contig from the 20 kb assembly was used as a bait to map back the ONT reads by Minimap2 v.2.19 [26], and the mapped reads were reassembled by Flye v. 2.9 in normal mode. For downstream analyses, we selected the assembly generated from the 10 kb-filtered reads, as we assumed that this read length would provide the best read coverage and repeat resolution after discovering that the genome contained a large repetitive region spanning over 5 kb. The resulting assembly was polished using POLCA v.4.0.9 [27].

### Genome assembly of ‘*Candidatus* Hepatoplasma vulgare Av-JP’

Length-filtered *A. vulgare* ONT reads were *de novo* assembled using metaFlye v. 2.9. A circular contig was identified using Bandage [28] and was used as a bait to map back the ONT reads by Minimap2. The mapped reads were then reassembled using Flye v. 2.9 and polished with Medaka v. 1.6.0 and POLCA v.4.0.9. Alignment of the reads revealed that the ribosomal RNA (rRNA) andtRNA cluster sequences of this assembly belonged to *Candidatus* Hepatoplasma crinochetorum Tokyo2021 (AP027132.1), which had higher sequencing coverage. As a result, we manually patched the corresponding regions using the Illumina assembly generated by SPAdes v. 3.15.3 [29], producing the finished assembly.

### Genome assembly of ‘*Candidatus* Hepatoplasma scabrum Ps-JP’

Filtered *P. scaber* Illumina reads were *de novo* assembled by SPAdes v. 3.15.3. The assembly contained a circular genome sequence, which was adopted as a MAG without any polishing. The Illumina reads were mapped against the assembly using Minimap2, and the alignment was visualized using Integrative Genomics Viewer (IGV) [30] to assess the integrity of the sequence. Although it is rather rare to recover a complete bacterial genome from Illumina reads alone, based on the read mapping results, we conclude that this contig represents a reasonably complete original genome sequence. Of note, this genome sequence was not recovered as a circular contig from the ONT assemblies due to low coverage.

### Genome assembly of ‘*Candidatus* Hepatoplasma crinochetorum Tokyo2021’

A circular contig was identified from the metaFlye assembly of *A. vulgare* ONT reads described above. The ONT reads were mapped back by Minimap2 and reassembled using Flye v. 2.9, followed by polishing with Medaka v. 1.6.0 and POLCA v.4.0.9.

### Genome annotation

The polished genome sequences were rotated to start at 100 bp upstream of the start codon of the *dnaA* gene. Given that each MAG was recovered as a complete and distinct genome, not a mixture of genomes from different organisms, we chose to annotate them using DFAST v. 1.2.18 [31]. This pipeline provided the annotated genomes submitted to INSDC via DDBJ. Additional functional annotations were performed for more in-depth analyses on metabolic capabilities (see ‘Reconstruction of metabolic pathways and exploration for digestive enzymes’). BUSCO v. 5.4.3 [32] was used to assess the completeness of the assembly based on the presence of single-copy genes conserved across *Mollicutes*. CheckM2 v. 1.0.1–0 [33] was also employed to independently gauge the assembly’s completeness and contamination level. CRISPR/Cas9-related proteins were identified by querying multiple sequence alignments (generated by MAFFT v. 7.505 [34]) of candidate homologs on the HHpred server (https://toolkit.tuebingen.mpg.de/tools/hhpred) [35]. Sequencing coverage was estimated by mapping ONT and Illumina reads against the MAGs using minimap2, followed by coverage calculation using SAMtools coverage [36]. Variant calling was performed using the Illumina BAM files by FreeBayes v. 1.3.6 [37]. Average nucleotide identities (ANI) and DNA–DNA hybridization values (DDH) were calculated using FastANI v. 1.33 [38] and Genome-to-Genome Distance Calculator (https://ggdc.dsmz.de/) [39], respectively.

### 16S metabarcoding of the shotgun sequencing data

We used phyloFlash v. 3.4.2 [40] and silva 138.1 database [41] to characterize the 16S ribosomal RNA gene (16S rDNA) sequences present in the filtered Illumina reads. The assembled 16S rDNA sequences were queried using NCBI blastn (https://blast.ncbi.nlm.nih.gov/Blast.cgi; last accessed October 2023) [42] against (i) the nonredundant nucleotide collection and (ii) 16S rRNA sequences from Bacteria and Archaea. Sequencing depths of the 16S rDNA contigs were calculated by mapping the 16S rDNA-associated reads extracted by phyloFlash onto the assembled 16S rDNA sequences using minimap2, followed by coverage calculation using SAMtools coverage.

### Phylogenetic analysis of 16S rDNA sequences

A total of 25 mollicute 16S rDNA sequences [12, 43] were downloaded from NCBI (accessed 12 October 2022) and aligned with MAFFT v. 7.505 [34] and trimmed using trimAl v. 1.4.1 [44]. The alignment was used for phylogenetic analysis with IQ-TREE v.2.2.0.3 [45]. A total of 484 DNA models and their combinations with additional parameters (F for frequencies, I for invariant sites, G for gamma distribution, and R for rate matrix variations), were tested using ModelFinder [46]. The best-fit model (GTR+F+R3) was selected based on Bayesian Information Criterion (BIC). Branch support was assessed using ultrafast bootstrap [47] with 1000 replicates. The resulting tree was visualized with FigTree v. 1.4.4 (http://tree.bio.ed.ac.uk/software/figtree/).

### Phylogenomic analysis

We used GTDB-tk v. 2.1.1 and GTDB-Tk reference data version r214 [48, 49] to confirm the phylogenetic positions of the MAGs based on the Genome Taxonomy Database (GTDB). Six *Hepatoplasmataceae* (‘f_Hepatoplasmataceae’) MAGs registered as GTDB species representatives were downloaded from NCBI (accessed August 2023), and protein-coding genes were predicted using Prodigal v. 2.6.3 [50]. The six GTDB-representative *Hepatoplasmataceae* proteomes, along with the proteomes of three MAGs characterized in this study, were combined with the proteomes (translated CDS) of 146 representative mollicute genomes downloaded from NCBI RefSeq (last accessed October 2022). The 146 representative mollicute genomes were derived from the type strains of their respective species, covering all major *Mollicute* families as delineated by Gupta *et al.* [51, 52]. A total of 155 mollicute proteomes were analysed with OrthoFinder v. 2.5.4 [53] to identify single-copy proteins universally conserved among mollicutes. Forty-one single-copy proteins were aligned with MAFFT v. 7.505, and the alignments were trimmed using trimAl v. 1.4.1. The trimmed alignments were used in maximum-likelihood phylogenetic analysis with IQ-TREE 2.2.0.3. A total of 132 protein models (LG, WAG, JTT, and their combinations with additional parameters F, I, G, and R) were tested using ModelFinder. The best-fit model (LG+F+I+I+R9) was selected based on BIC. Branch support was assessed using ultrafast bootstrap with 1000 replicates. The phylogenetic tree was visualized with FigTree.

### Reconstruction of metabolic pathways and exploration for digestive enzymes

The KEGG pathway maps of four *Hepatoplasmatacae* MAGs characterized in this study were constructed on the BLASTKOALA server (https://www.kegg.jp/blastkoala/) [54]. The results were visualized on the KEGG Mapper server (https://www.kegg.jp/kegg/mapper/) [55] and used as a reference to build the schematic map of metabolic pathways in Fig. 4, which was drawn manually. Genes missing from the original KEGG outputs were manually identified with the aid of homology searches using BLASTP, HHpred, and HMMER3 [[56]]. Carbohydrate-degrading enzymes were searched on the dbCAN2 meta server (https://bcb.unl.edu/dbCAN2/) [57].

### Exploration of proteins possibly related to host-symbiont associations

We used antiSMASH v. 6.1.1 [58] and NaPDoS2 server (https://npdomainseeker.sdsc.edu/napdos2/) [59] to exhaustively screen for gene clusters related to secondary metabolite production. We used InterProScan v. 5.64–96.0 [60] to search for nucleases, proteases, and lipases present in the four *Hepatoplasmataceae* MAGs. From the from the InterProScan outputs, we extracted lines that matched keywords such as 'nuclease,' 'peptidase', 'protease,' or 'lipase' using grep and saved the corresponding protein IDs. The candidate nucleases, peptidases, and lipases from four proteomes were clustered using MMSeqs2 easy-cluster [61], aligned with MAFFT, and queried on the HHpred server [35] against Pfam-A_v36 [62], NCBI_Conserved_Domains(CD)_v3.19 [63], SMART_v6.0 [64], and TIGRFAMs_v15.0 [65] databases, and the outputs were inspected manually. Eukaryotic-like proteins (ELPs) were explored using EffectiveELD search on the EffectiveDB server (https://www.effectivedb.org/method/effectiveeld) [66]. Three-dimensional (3D) structure predictions were performed on the ColabFold v1.5.2 notebook (https://colab.research.google.com/github/sokrypton/ColabFold/blob/main/AlphaFold2.ipynb) [67, 68]. The 3D models were visualized using ChimeraX [69]. Repetitive motifs in protein sequences were identified using MEME v. 5.5.3 [70]. Sequence logos were drawn using WebLogo v. 3.7.9 [71].

## Results

### Metagenome-assembled genome sequences of novel Hepatoplasma relatives

We generated 11.0 to 39.6 Gb of ONT reads and 21.3 to 23.8 Gb of 2×150 bp Illumina paired-end reads (Table 1). From these shotgun sequence data, we recovered four MAGs representing isopod-associated mollicutes (Table 2). *Candidatus* Tyloplasma litorale Fukuoka2020 (AP027078.1) was likely the dominant bacterial symbiont of the *T. granuliferus* animal analysed, as it was the only genome that was successfully assembled from the ONT reads. We recovered complete MAGs of *Candidatus* Hepatoplasma crinochetorum Tokyo2021 (AP027132.1) and *Candidatus* Hepatoplasma vulgare Av-JP (AP027131.1) from *A. vulgare* ONT reads. The metaFlye assembly of *A. vulgare* also contained a *Paracoccus*-like genome and a *Rickettsia*-like genome. *Candidatus* Hepatoplasma scabrum Ps-JP (AP027133.1) was recovered as a circular contig from the Illumina assembly of *P. scaber* reads.

**Table 1.** Sequencing statistics of isopod hologenomes

Species		*Armadillidium vulgare*	*Porcellio scaber*	*Tylos granuliferus*
**Isolate**		TUMSAT20210906	TUMSAT20211004	Kyushu2020*
**Sampling date**		2021/9/6	2021/10/4	2020
**Sampling location**		Japan:Tokyo	Japan:Tokyo	Japan:Kyushu*
**Latitude and longitude**		35.62579081 N 139.74790018 E	35.62558752 N 139.75034157 E	n/a†
**BioSample**		SAMD00511446	SAMD00511447	SAMD00511451
**ONT**	**Run**	DRR394929	DRR394930	DRR394945
	**Number of reads**	1 561 246	1 788 627	11 362 452
	**Total bases**	14 106 876 463	11 038 910 306	39 629 071 812
	**Read length N50**	13 812	9193	5686
**Illumina**	**Run**	DRR394921	DRR394922	DRR394944
	**Number of reads**	141 310 158	149 921 108	81 448 691
	**Total bases**	21 337 833 858	22 638 087 308	24 597 504 682

*Fukuoka Prefecture in Kyushu Island.

†N/A: not available.

**Table 2.** Genome assembly statistics of *Hepatoplasmataceae* representatives

Species		*Candidatus* Tyloplasma litorale	*Candidatus* Hepatoplasma vulgare	*Candidatus* Hepatoplasma scabrum	*Candidatus* Hepatoplasma crinochetorum	
Isolate		Fukuoka2020	Av-JP	Ps-JP	Tokyo2021	Av*
Reference		This study	This study	This study	This study	[2, 6]
Host		*Tylos granuliferus*	*Armadillidium vulgare*	*Porcellio scaber*	*Armadillidium vulgare*	*Armadillidium vulgare*
Accession no.		AP027078.1	AP027131.1	AP027133.1	AP027132.1	NZ_CP006932.1
Length (bp)		615 622	662 079	606 194	643 039	657 101
GC content (%)		24.4	22.7	24.7	22.6	22.5
CDSs		530	597	543	573	577
rRNAs		6	3	3	3	3
tRNAs		25	26	28	27	28
CRISPRs		2	1	1	1	1
BUSCO completeness	Complete BUSCOs (C)	158 (90.8 %)	158 (90.8 %)	156 (89.7 %)	152 (87.4 %)	152 (87.4 %)
[mycoplasmatales_odb10 (*n*=174), genome mode]	Complete and single-copy BUSCOs (S)	158 (90.8 %)	158 (90.8 %)	156 (89.7 %)	152 (87.4 %)	152 (87.4 %)
	Complete and duplicated BUSCOs (D)	0 (0.0 %)	0 (0.0 %)	0 (0.0 %)	0 (0.0 %)	0 (0.0 %)
	Fragmented BUSCOs (F)	1 (0.6 %)	3 (1.7 %)	2 (1.1 %)	3 (1.7 %)	3 (1.7 %)
	Missing BUSCOs (M)	15 (8.6 %)	13 (7.5 %)	16 (9.2 %)	19 (10.9 %)	19 (10.9 %)
CheckM2 (%)	Completeness	93.66	90.16	96.53	96.31	96.87
	Contamination	0.09	0.5	0.0	0.01	0.05
Coverage	Illumina	527.422	38.2049	19.997	761.155	–
	ONT (5 kb>)	223.069	14.9362	4.92264	298.474	–
	Illumina SVs	10	42	30	1	–

*Shown for comparison.

**Fig. 1.**
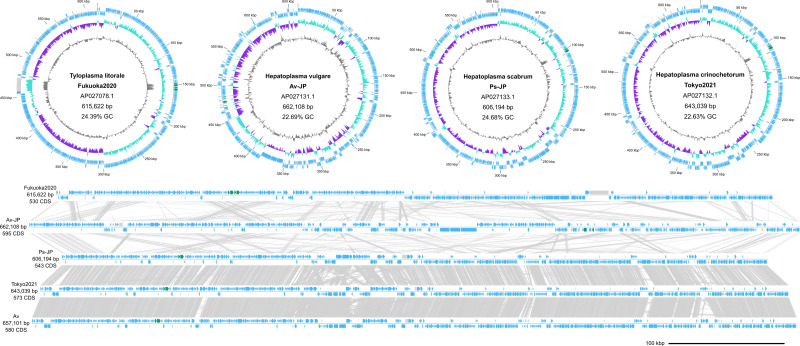
Genome diagrams of *Hepatoplasmataceae* members. (**a**) Circular genome diagrams of *Hepatoplasmataceae* members. Arrowheads indicate the transcriptional orientation. Outer track: protein-coding genes (blue), ribosomal RNA genes (green), transfer RNA genes (orange), repeat regions (grey). Middle track: GC skew of 100 bp sliding windows with 10 bp increments (positive: emerald, negative: purple). Inner track: Deviation of GC contents from the average, 100 bp sliding windows with 10 bp increments. (**b**) Linear diagrams of *Hepatoplasmataceae* genomes. The reference genome for ‘*Candidatus* Hepatoplasma crinochetorum’ is shown at the bottom as isolate ‘Av’ for comparison. TBLASTX hits (e-value: 1e-3, bitscore:50) are shown in grey.

The four *Hepatoplasmataceae* MAGs ranged in size from 606 kb to 662 kb and had GC contents of 22.6–24.4 % (Fig. 1, Table 2). Small genome sizes and low GC contents are characteristic to mollicute genomes. A range of 530 to 597 protein-coding genes were detected in the recovered MAGs. Illumina read alignment detected an average of 20.8 structural variants per MAG, suggesting that the assembled MAGs represent clonal populations.

We first used BUSCO [32] to analyse the completeness of the MAGs, because they were regarded as virtually equivalent to the complete genomes of bacterial isolates. The BUSCO completeness scores for the mycoplasmatales_odb10 dataset in genome mode ranged from 87.4–90.8 %. Although these scores are somewhat low, this is most likely due to real gene loss or extensive sequence divergence, rather than assembly incompleteness, as the complete genome of *Candidatus* Hepatoplasma crinochetorum Av (NZ_CP006932.1) had similar BUSCO values (Table 2). We also used CheckM2 [33] to assess the completeness and the level of contamination of the assembled MAGs. The CheckM2 completeness scores ranged from 90.16–96.53% and showed little sign of contamination (0.0–0.5 %), values comparable to the *Candidatus* Hepatoplasma crinochetorum Av genome (completeness: 96.87 %; contamination: 0.05 %). Overall, these results indicate that the *Hepatoplasmataceae* MAGs assembled in this study are of high quality and most likely to be complete.

We used phyloFlash [40] to estimate the taxonomic compositions of rDNA sequences in the Illumina reads (Tables S1 and S2). Eukaryotic rDNA sequences were the most abundant in all three datasets, reflecting the fact that they are shotgun sequencing data of the isopod hosts (Table S1). The *P. scaber* library contained a non-isopod eukaryotic 18S rDNA (DRR394922.PFspades_2_34.308066; Table 3; DRR394922.all.final.fasta, phyloflash.zip); this exhibited 99–100 % nucleotide identity to the 18S rDNA sequences of Acanthocephalan parasites *Plagiorhynchus* spp. (e.g. AF001839.1 and MN057694.1), which have been known to infect isopods as an intermediate host [72]. The *A. vulgare* library contained two distinct *Hepatoplasmataceae* sequences of markedly different read depths (*Candidatus* Hepatoplasma crinochetorum Tokyo2021: DRR394921.PFspades_2_81.254492; *Candidatus* Hepatoplasma vulgare Av-JP: DRR394921.PFspades_6_3.617203; Table S2; DRR394921.all.final.fasta in PhyloFlash.zip), in agreement with the presence of two distinct *Hepatoplasmataceae* MAGs in the ONT assembly. *Candidatus* Tyloplasma litorale (DRR394944.PFspades_2_99.178834; Table S2; DRR394944.all.final.fasta, PhyloFlash.zip) was the dominant bacterial species in the *T. granuliferus* library, in line with the absence of bacterial contigs other than that of *Candidatus* Tyloplasma litorale in the metaFlye assembly. No *Wolbachia-*like sequences were detected in any of the three datasets.

**Table 3.** Comparative genome similarity metrics of *Hepatoplasmataceae* MAGs

DDH (%)	Av	Tokyo2021	Ps	Ps-JP	Av-JP	Fukuoka2020	DT_51	GLR43	Bg1	Bg2	DT_50
Av	100	85.7	22	20.9	18.2	17.6	16	16.9	15.8	14.9	0
Tokyo2021	85.7	100	22.1	20.9	18.6	17.6	16.1	16.9	15.9	14.8	0
Ps	22	22.1	100	24	23.5	22.9	16.4	20	15.9	17.9	0
Ps-JP	20.9	20.9	24	100	20.4	20.6	14.9	19.1	16.1	17.8	0
Av-JP	18.2	18.6	23.5	20.4	100	25	16.8	15	16.3	16.8	0
Fukuoka2020	17.6	17.6	22.9	20.6	25	100	17.4	18.4	17.8	17.1	20.3
DT_51	16	16.1	16.4	14.9	16.8	17.4	100	16	14.1	18.9	18
GLR43	16.9	16.9	20	19.1	15	18.4	16	100	15.7	16	0
Bg1	15.8	15.9	15.9	16.1	16.3	17.8	14.1	15.7	100	24.7	0
Bg2	14.9	14.8	17.9	17.8	16.8	17.1	18.9	16	24.7	100	0
DT_50	0	0	0	0	0	20.3	18	0	0	0	100
**ANI (%)**	**Av**	**Tokyo2021**	**Ps**	**Ps-JP**	**Av-JP**	**Fukuoka2020**	**DT_51**	**GLR43**	**Bg1**	**Bg2**	**DT_50**
Av	100	98.4117	82.45	81.288	76.676	76.2394	0	0	0	0	0
Tokyo2021	98.43	100	82.67	81.408	76.758	76.3795	75.74	0	0	0	0
Ps	82.8	82.6703	100	83.034	76.191	76.1979	0	0	75.61	0	0
Ps-JP	81.24	81.4081	83.12	100	76.526	0	0	0	0	0	0
Av-JP	76.57	76.7301	76.07	76.203	100	76.064	0	0	0	0	0
Fukuoka2020	76.14	76.3752	75.97	0	76.002	100	78.13	0	0	0	0
DT_51	0	75.7442	0	0	75.641	78.7044	100	0	0	0	0
GLR43	0	0	0	0	0	0	0	100	77.08	76.5	0
Bg1	0	0	75.61	0	0	0	0	76.902	100	84.2	0
Bg2	0	0	0	0	0	0	0	0	84.29	100	0
DT_50	0	0	0	0	0	0	0	0	0	0	100
**AAI (%)**	**Av**	**Tokyo2021**	**Ps**	**Ps-JP**	**Av-JP**	**Fukuoka2020**	**DT_51**	**GLR43**	**Bg1**	**Bg2**	**DT_50**
Av	>90	>90	61.93	61.44	46.91	46.8	46.46	48.52	46.31	46.2	41.66
Tokyo2021	>90	>90	61.9	61.4	46.92	46.8	46.47	48.48	46.29	46.2	41.65
Ps	61.93	61.9	>90	64.09	46.96	46.29	46.22	48.33	46.45	46.2	41.61
Ps-JP	61.44	61.4	64.09	>90	46.66	46.57	46.32	48.35	46.55	46.4	41.68
Av-JP	46.91	46.92	46.96	46.66	>90	45.51	45.2	46.98	44.92	44.9	41.03
Fukuoka2020	46.8	46.8	46.29	46.57	45.51	>90	53.94	49.56	47.51	47.8	41.73
DT_51	46.46	46.47	46.22	46.32	45.2	53.94	>90	48.89	47.11	47.1	41.25
GLR43	48.52	48.48	48.33	48.35	46.98	49.56	48.89	>90	53.78	53.5	43.29
Bg1	46.31	46.29	46.45	46.55	44.92	47.51	47.11	53.78	>90	66.4	41.52
Bg2	46.2	46.18	46.24	46.36	44.92	47.83	47.11	53.48	66.44	>90	41.33
DT_50	41.66	41.65	41.61	41.68	41.03	41.73	41.25	43.29	41.52	41.3	>90

DDH: DDH values calculated by Genome-to-Genome Distance Calculator (Formula 2: identities / HSP length); ANI: ANI values calculated by FastANI; AAI: AAI values calculated by FastAAI**.** Note: Entries denoted ‘>90%’ reflect an AAI above this threshold; exact values between 90% and 100% are not specified due to FastAAI's rounding. Av: *Candidatus* Hepatoplasma crinochetorum Av (NZ_CP006932.1); Tokyo2021: *Candidatus* Hepatoplasma crinochetorum Tokyo2021 (AP027132.1); Ps: Hepatoplasma crinochetorum Ps (GCA_001179805.1); Ps-JP: *Candidatus* Hepatoplasma scabrum Ps-JP (AP027133.1); Av-JP: *Candidatus* Hepatoplasma vulgare Av-JP (AP027131.1); Fukuoka2020: *Candidatus* Tyloplasma litorale Fukuoka2020 (AP027078.1); DT_51: DT_51 (GCA_013214765.1); GLR43: GLR43 (GCA_013139135.1); Bg1: Bg1 (GCA_001641205.1); Bg2: (GCA_001641225.1); DT_50: DT_50 (GCA_013214845.1).

**Fig. 2.**
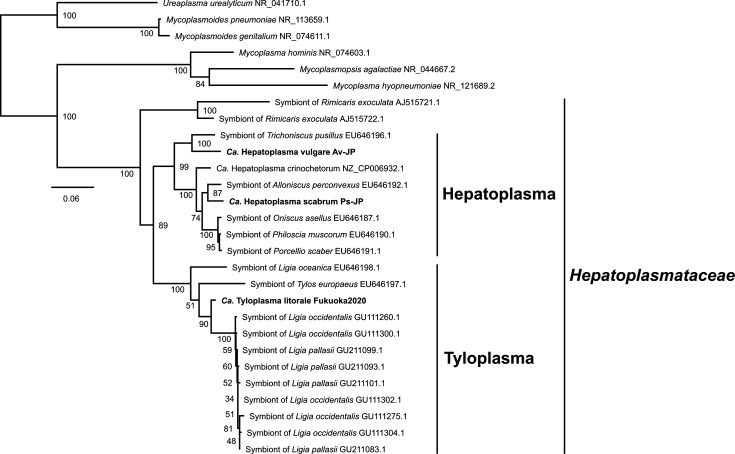
Phylogenetic analysis of 16S rDNA gene sequences. A total of 1462 sites (model: GTR+F+R3) were used in the maximum-likelihood phylogenetic analysis using IQ-TREE v. 2.2.0.3. Values beside nodes indicate the ultrafast bootstrap support (1000 trials).

A maximum-likelihood phylogenetic tree based on 16S rDNA sequences placed *Candidatus* Tyloplasma litorale Fukuoka2020 into a monophyletic clade consisting of semiterrestrial isopod-associated mollicutes (Fig. 2). This clade was a sister clade of the terrestrial isopod-associated mollicutes, including *Candidatus* Hepatoplasma crinochetorum and its closest relatives.

We used GTDB-tk [48] to place the MAGs in the currently known diversity of bacterial MAGs. The analysis placed all MAGs into *Hepatoplasmatacae*, confirming their taxonomic position at the family level (Table S3). *Candidatus* Tyloplasma litorale Fukuoka2020 fell into genus g_Bg2, a placeholder taxon represented by four MAGs including Bg2 (GCA_001641225.1), which was identified from the deep-sea isopod *Bathynomus* sp. [21]. *Candidatus* Hepatoplasma crinochetorum Tokyo2021 was unambiguously classified into *Candidatus* Hepatoplasma crinochetorum, while *Candidatus* Hepatoplasma vulgare Av-JP and *Candidatus* Hepatoplasma scabrum Ps-JP were flagged as novel species in *Candidatus* Hepatoplasma.

**Fig. 3.**
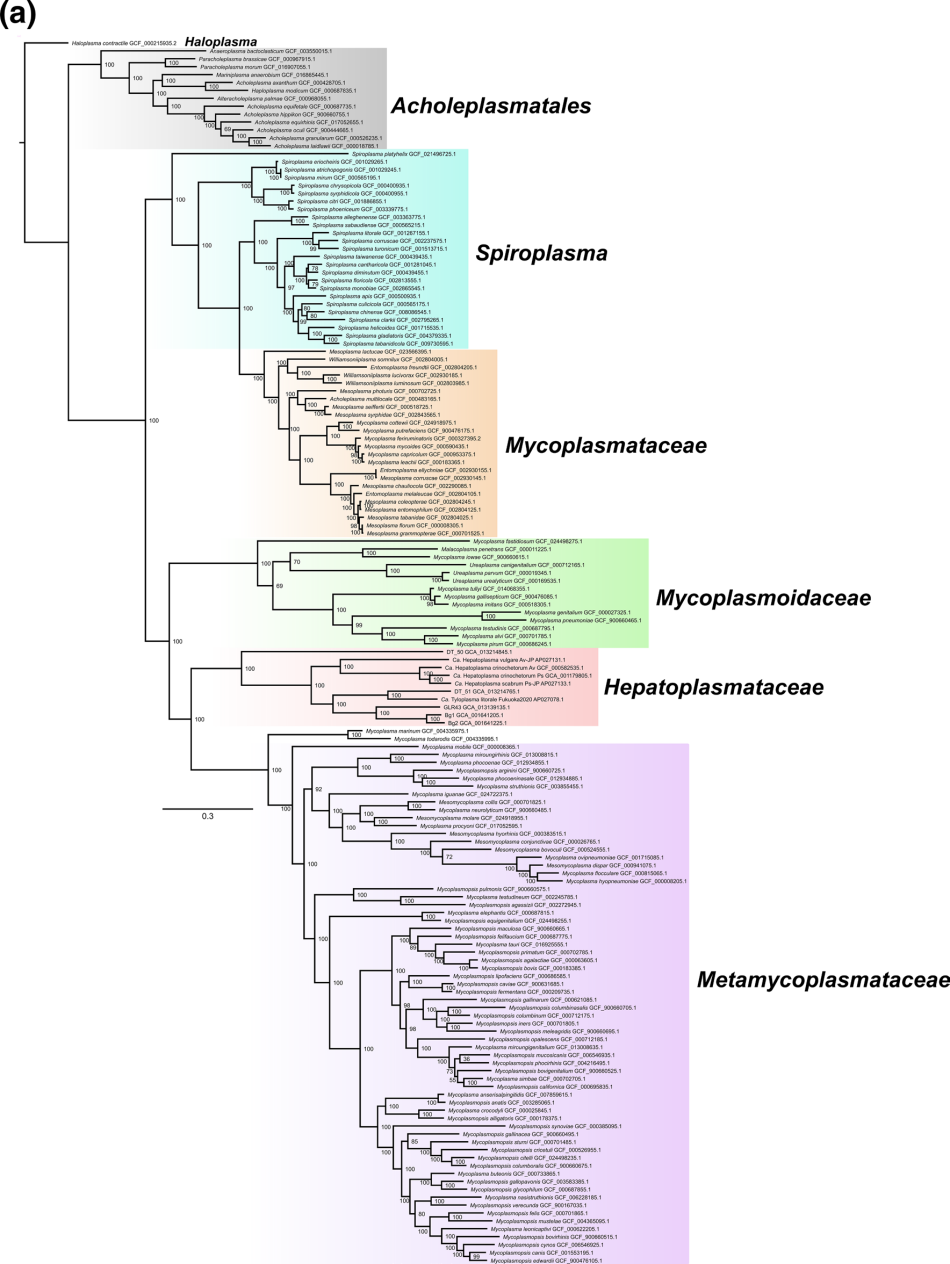

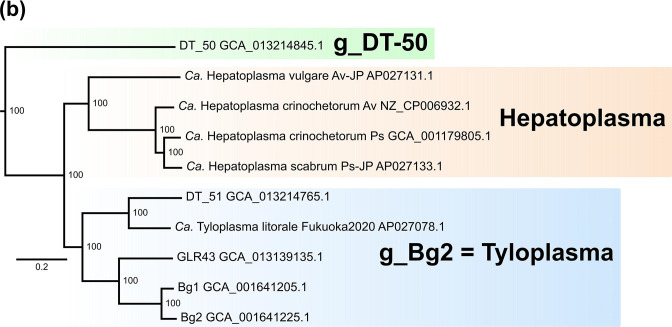
Phylogenomic analysis of *Mollicutes.* (**a**) A total of 41 single-copy proteins conserved among 155 mollicute genomes (8219 sites; model: LG+F+I+I+R10) were used in the maximum-likelihood phylogenetic analysis using IQ-TREE v. 2.2.0.3. Values beside nodes indicate the ultrafast bootstrap support (1000 trials). Clades were delineated according to Gupta *et al.* [51, 52]. (**b**) Subtree of (**a**) highlighting *Hepatoplasmataceae.* Names in bold indicate genus names in the GTDB-Tk reference data version r214 [48, 49].

To further investigate the phylogenetic position of *Hepatoplasmataceae* and the relationships of isopod-associated mollicutes within the family, we built a maximum-likelihood phylogenomic tree of 155 mollicutes based on 41 single-copy protein-coding genes identified by OrthoFinder [53] (Fig. 3). The resulting tree recovered *Hepatoplasmataceae* as a sister clade of *Metamycoplasmataceae* (formerly known as the Bovis group) [52] (Fig. 3a). Within the *Hepatoplasmataceae* family were three major branches, which largely agree with genus demarcations in GTDB r214 release [49]: *Candidatus* Hepatoplasma and two placeholder genera, g_Bg2 and g_DT-50 (Fig. 3b). *Candidatus* Hepatoplasma crinochetorum Tokyo2021, *Candidatus* Hepatoplasma vulgare Av-JP, and *Candidatus* Hepatoplasma scabrum Ps-JP fell into *Candidatus* Hepatoplasma, while *Candidatus* Tyloplasma litorale Fukuoka2020 clustered with g_Bg2. All MAGs currently designated as g_Bg2 in the r214 release of GTDB (DT_51, GLR43, Bg1, and Bg2) are short read-based draft assemblies and lack 16S rDNA sequences. Consequently, the complete MAG of *Candidatus* Tyloplasma litorale Fukuoka2020, featuring two copies of entire rDNA clusters, serves as a connection between g_Bg2-like MAGs missing 16S rDNA and 16S rDNA sequences found solely in barcode sequences from semiterrestrial isopods.

ANI and DDH values are widely used to delineate bacterial species based on sequence similarities [73, 74]. The ANI and DDH values of *Hepatoplasmataceae* MAGs assembled in this study were substantially lower than same species thresholds (95 % for ANI and 70 % for DDH) for any combinations except for the pair *Candidatus* Hepatoplasma crinochetorum Toyko2021 and *Candidatus* Hepatoplasma crinochetorum Av (Table 3). This again indicates that three *Hepatoplasmataceae* MAGs represent distinct bacterial species within *Hepatoplasmataceae* family.

The *Hepatoplasmataceae* MAGs characterized in this study showed high completeness and little signs of contamination. Their placement within the family was verified through genome-based taxonomy using GTDB-tk and 16S rDNA sequences. The ANI and DDH values for three of the MAGs, *Candidatus* Tyloplasma litorale Fukuoka2020, *Candidatus* Hepatoplasma vulgare Av-JP, and *Candidatus* Hepatoplasma scabrum Ps-JP, were significantly below the thresholds for existing species classification, as shown in Table 3. Hence, these three MAGs fulfil the criteria for being designated as the nomenclatural types for their corresponding species names, as outlined in the SeqCode initiative [75].

We propose the name *Candidatus* Tyloplasma for the GTDB placeholder genus g_Bg2, acknowledging its first identification as uncultured mollicutes from semi-terrestrial isopods (*Tylos europeaus* and *Ligia occidentalis*) [12], and the discovery of its first complete MAG from *Tylos granuliferus*. We also introduce two novel genomospecies *Candidatus* Hepatoplasma vulgare and *Candidatus* Hepatoplasma scabrum. These genomospecies are distinguished by their low average nucleotide identities compared to other members of the *Hepatoplasmataceae* family. *Candidatus* Hepatoplasma scabrum is the closest relative of *Candidatus* Hepatoplasma crinochetorum identified so far, characterized by an extensive collinearity throughout the genome (Fig. 1b).

### *Hepatoplasmataceae* retains an intact type IIA CRISPR-Cas9 system

We identified up to three phage defence mechanisms in the *Hepatoplasmataceae* MAGs: type I and II restriction modification systems and a likely intact type IIA CRISPR-Cas9 system (Table 4). All three defence systems were found on the *Candidatus* Tyloplasma litorale genome, whereas the type I restriction modification system was absent or incomplete in *Candidatus* Hepatoplasma vulgare and *Candidatus* Hepatoplasma scabrum. The initial report on the *Candidatus* Hepatoplasma crinochetorum genome suggested that the CRISPR/Cas machinery is no longer functional due to the loss of the helper protein Csn2 [18]. However, using HHpred, we found Csn2 homologs in the vicinities of the CRISPR arrays. This indicates that *Hepatoplasmataceae* is equipped with a complete set of Type IIA CRISPR/Cas9 machinery.

**Table 4.** Genome defense-related genes in *Hepatoplasmataceae*

Functional category	Protein	*Candidatus* Tyloplasma litorale	*Candidatus* Hepatoplasma vulgare	*Candidatus* Hepatoplasma scabrum	*Candidatus* Hepatoplasma crinochetorum
Type I restriction endonuclease	subunit S	BDU67866.1 BDU67868.1 BDU67869.1	–	–	BDV02943.1
	subunit R	BDU67870.1	–	–	BDV02944.1
	subunit M	BDU67867.1	BDV02406.1	–	BDV02945.1
Type II restriction endonuclease	MjaI	BDU67724.1	–	–	–
	SauIIIAI	–	BDV02278.1	–	–
	DpnI	BDU67733.1	–	–	–
Type II CRISPR/Cas9	Cas1	BDU67747.1	BDV02176.1	BDV03524.1	WP_025208688.1
	Cas2	BDU67748.1	BDV02174.1	BDV03523.1	WP_128571630.1
	Cas9	BDU67745.1	BDV02189.1	BDV03530.1	WP_025208688.1
	Csn2	BDU67749.1	BDV02173.1	BDV03522.1	WP_025208679.1

### Nutritional dependence on the host

**Fig. 4.**
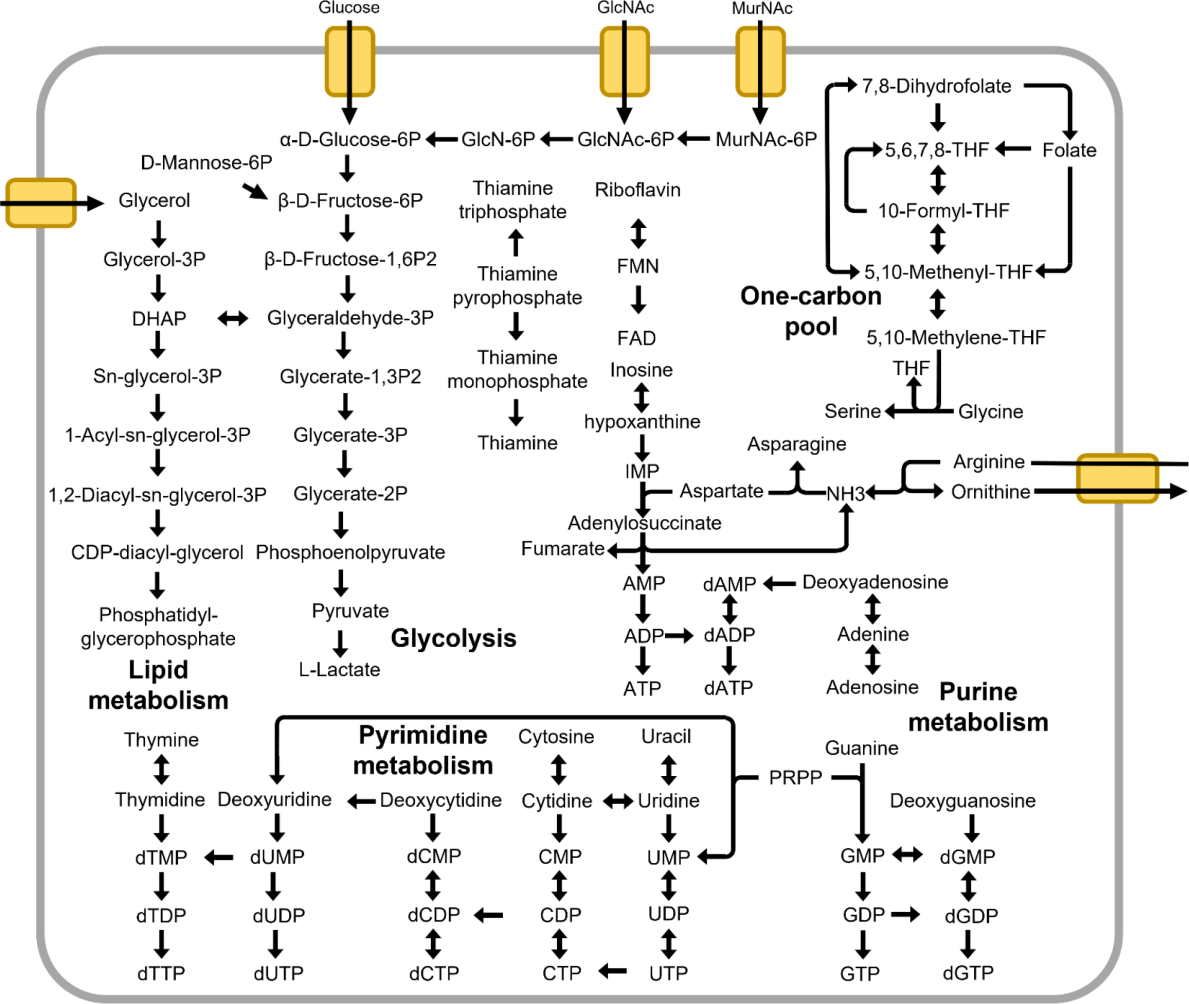
Summary of metabolic pathways in *Hepatoplasmataceae.* The diagram was drawn manually based on KEGG Pathway diagrams generated on the BLASTKOALA server (https://www.kegg.jp/blastkoala/) (53), incorporating manually curated genes in Table 5.

*Hepatoplasmataceae*, like other mollicutes, lack many of the biosynthetic pathways necessary for the production of amino acids, nucleic acids, and carbohydrates. Instead, these pathways are likely substituted by various transport proteins, such as ABC transporters [76, 77] and the phosphoenolpyruvate (PEP): carbohydrate phosphotransferase system (PTS) [78, 79]. A summary of *Hepatoplasmataceae* metabolic pathways is shown in Fig. 4.

*Hepatoplasmataceae* are only able to catabolize carbohydrates through glycolysis. The PTS catalyses the uptake and concomitant phosphorylation of carbohydrates in bacteria [78, 79]. *Candidatus* Tyloplasma litorale Fukuoka2020 encode PTS systems for five sugars: glucose, fructose, trehalose, *N*-Acetyl-d-glucosamine (GlcNAc), and *N*-Acetyl-muramic acid (MurNAc), while *Candidatus* Hepatoplasma spp. lacked the PTS for GlcNAc (Table 3). A mannose isomerase was present in all four *Hepatoplasmataceae* MAGs, suggesting the ability to utilize mannose, but we could not identify a mannose transporter protein. The sugars are converted to beta-d-fructose 6-phosphate and enter the glycolysis pathway (Fig. 4). The ability to metabolize GlcNAc and MurNAc, the building blocks of bacterial cell walls, means that *Hepatoplasmataceae* can utilize debris from other cell wall-containing bacteria.

*Hepatoplasmataceae* lack most of the amino acid synthesis pathways and therefore must import them from the environment through as-yet unknown transporters. The conversion of glycine to serine by serine hydroxymethyltransferase (EC 2.1.2.1) is coupled with the conversion of 5,10-methylenetetrahydrofolate to tetrahydrofolate, which is part of the one-carbon pool. Aspartate is converted to asparagine by the aspartate-ammonia ligase (EC 6.3.1.1). The ammonia moiety could be derived from the purine nucleotide cycle in the nucleotide savage pathway and the arginine deaminase (ADI) pathway (discussed below).

The ADI pathway, composed of arginine deiminase (EC 3.5.3.6), carbamate kinase (EC 2.7.2.2), and arginine/ornithine antiporter, produces ATP (adenosine triphosphate) through the conversion of arginine to citrulline to ornithine [80] (Table 5). This pathway has been suggested to act as a pH buffer in order to counteract acidification resulting from glycolysis [19, 21]. A complete set of ADI pathway components was present in *Candidatus* Hepatoplasma spp., whereas *Candidatus* Tyloplasma litorale lacked them altogether. The presence of the ADI pathway has been reported in other *Hepatoplasma* draft MAGs [19, 21]. This suggests that the absence of the ADI pathway in *Candidatus* Tyloplasma litorale is due to a secondary loss.

**Table 5.** Examples of metabolism-related genes in *Hepatoplasmataceae*

Functions	Description	Gene	Candidatus Tyloplasma litorale	Candidatus Hepatoplasma vulgare	Candidatus Hepatoplasma scabrum	Candidatus Hepatoplasma crinochetorum
Glycerol utilization	Aquaporin	–	BDU67486.1	BDV02492.1, BDV02493.1	BDV03539.1	WP_025208695.1
	Glycerol uptake facilitator protein	*glpF*	–	BDV02358.1	BDV03527.1	WP_025208684.1
	Glycerol kinase	*glpK*	–	BDV02359.1	BDV03528.1	WP_025208685.1
	Glycerol-3-phosphate dehydrogenase	*glpA*	–	BDV02360.1	BDV03529.1	WP_128571633.1
Arginine deiminase pathway	Arginine deiminase	*arcA*	–	BDV02199.1	BDV03492.1	WP_038462236.1
	Ornithine carbamoyltransferase, catabolic	*arcB*	–	BDV02200.1	BDV03493.1	WP_025208634.1
	Carbamate kinase	*arcC*	–	BDV02202.1	BDV03495.1	WP_025208637.1
	Arginine/ornithine antiporter	*arcD*	–	BDV02201.1	BDV03494.1	WP_025208636.1
PTS	Trehalose transporter	–	BDU67689.1	BDV02506.1	BDV03526.1	WP_025208683.1
	Glucose transporter	–	BDU67775.1	BDV02499.1	BDV03671.1	WP_025208828.1
	GlcNAc transporter	–	BDU67558.1	BDV02647.1	BDV03283.1	WP_025208424.1
	MurNAc transporter	–	BDU67422.1	–	–	–
	Fructose transporter	–	BDU67439.1	BDV02545.1	BDV03409.1	WP_025208549.1

Mycoplasmas are able to synthesize glycerophospholipids, the main components of the cell membrane [81]. Most of the enzymes required for the biosynthesis of glycerophospholipids were successfully identified, but the phosphatidylglycerophosphatase (EC:3.1.3.27) was absent from the *Hepatoplasmataceae* MAGs. The lack of this gene in *Mycoplasma* genomes has been noted in the comparative genomic analyses of swine respiratory tract mycoplasmas [82]. As the authors of [82] noted, this enzymatic reaction should be present and is likely to be replaced by other gene(s).

Glycerol utilization has been linked to the virulence of *Mycoplasma* [81, 83]. An aquaporin protein gene, which could functions as a glycerol importer [84], was identified in all four *Hepatoplasmataceae* MAGs (Table 5). Additionally, *Candidatus* Hepatoplasma genomes encoded a gene cluster associated with glycerol utilization and therefore are likely able to utilize glycerol as carbon source, while *Candidatus* Tyloplasma litorale lacks components of this pathway.

As with other mollicutes, *Hepatoplasmataceae* lack a *de novo* synthesis pathway for nucleotides and therefore rely on import and the salvage pathway. Purine nucleobases are converted to nucleosides by purine nucleoside phosphorylase (EC 2.4.2.1), and then to nucleotide by deoxyadenosine kinase (EC 2.7.1.76). *Hepatoplasmataceae* and *Mycolasma pneumoniae* encode deoxyadenosine/deoxycytidine kinase (EC:2.7.1.76 2.7.1.74), but not deoxyguanosine kinase (DGK; EC 2.7.1.113), even though DGK activity has been detected in *M. pneumoniae* [85]. Hypoxanthine is converted to inositol monophosphate (IMP) by hypoxanthine phosphoribosyltransferase (EC:2.4.2.8). IMP is converted to adenosine monophosphate in the purine nucleotide cycle. However, xanthine dehydrogenase/oxidase (EC:1.17.1.4 1.17.3.2) and guanine deaminase (EC:3.5.4.3) were missing from *Hepatoplasmataceae* genomes, suggesting that the interconversion between purine bases is not possible [86, 87]. In addition, nucleoside-diphosphate kinase (EC:2.7.4.6) was missing from *Hepatoplasmataceae* and other mollicute genomes, which is likely to be compensated for by monophosphate kinases [88].

*Hepatoplasmataceae* lack cofactor synthesis pathways and therefore are unlikely to contribute these nutrients to the host. *Hepatoplasmataceae* MAGs lacked nicotinamide phosphoribosyltransferase (EC:2.4.2.12) and nicotinamide adenine dinucleotide kinase kinase (EC:2.7.1.23), both of which are present in *M. pneunmoniae*. Flavin mononucleotide (FMN) and derivatives can potentially be imported by specific transporters, which seem to be uniquely expanded in *Candidatus* Tyloplasma litorale (BDU67363.1, BDU67639.1, BDU67349.1, BDU67732.1, and BDU67420.1). Riboflavin is converted to FMN and then to flavin adenine dinucleotide. Thiamine kinase (EC 2.7.1.89), thiamine-monophosphate kinase (EC 2.7.4.16), and thiamine pyrophosphokinase (EC 2.7.6.2) were not detected in the *Hepatoplasmataceae* MAGs. The one-carbon pool seems to be functional in *Hepatoplasmataceae*.

ABC transporters represent the largest group of active membrane transport proteins in bacteria [83, 89]. *Candidatus* Tyloplasma litorale Fukuoka2020 encoded at least five ABC transporter systems. The substrate molecules could not be determined based on homology search due to ambiguous search outcome.

Overall, *Hepatoplasmataceae* are highly dependent on the host for nutrition and are unlikely to code for biosynthetic pathways providing essential nutrients to the host. A few differences in metabolic pathways exist among the four *Hepatoplasmataceae* species; while *Candidatus* Tyloplasma litorale Fukuoka2020 seems to be slightly more versatile in terms of its ability to utilize various sugars, *Candidatus* Hepatoplasma spp. seem to exploit the arginine deaminase pathway as a means of generating ATP.

### Limited evidence of enzymes providing nutritional benefits to the host

Hepatoplasmas have been suggested to be a nutritional symbiont that aid the digestion, especially that of polysaccharides [12, 14, 43]. We searched for genes encoding polysaccharide-degrading enzymes in *Hepatoplasmataceae* MAGs using dbCAN2 [57]. The only carbohydrate-degrading enzyme found was an alpha,alpha-phosphotrehalase, a member of glycoside hydrolase family 13 (GH13), in *Candidatus* Hepatoplasma vulgare (BDV02505.1), *Candidatus* Hepatoplasma scabrum (BDV03525.1) and *Candidatus* Hepatoplasma crinochetorum (WP_025208682.1 and BDV02972.1). No carbohydrate-degrading enzyme was found in *Candidatus* Tyloplasma litorale. These observations strongly suggest that *Hepatoplasmataceae* do not possess enzymes related to polysaccharide degradation.

To identify other types of enzymes possibly related to nutritional symbiosis, we analysed the *Hepatoplasmataceae* proteomes using InterProScan. This search identified multiple nucleases, proteases/peptidases, and a lipase (Table S4).

A range of 28 to 31 nuclease domain-containing proteins were detected in the *Hepatoplasmataceae* proteomes. Apart from conserved proteins involved with DNA and RNA metabolism, we also identified up to three copies per MAG of GIY-YIG and HNH nucleases with unknown functions, suggesting the potential for specialized roles.

The *Hepatoplasmatacae* MAGs encoded between nine to twelve peptidases. In addition to highly conserved genes such as ribosomal-processing cysteine protease Prp, ATP-dependent metalloprotease FtsH, and ATP-dependent serine endopeptidase La, five to seven peptidases of ambiguous physiological importance were identified.

The only lipase found in the four *Hepatoplasmataceae* MAGs was a patatin-like phospholipase. This protein was universally conserved across the four MAGs, implying functional importance. However, BLASTP searches against the NCBI non-redundant protein database returned numerous hits from non-mollicute bacteria but few positive hits from other mollicutes, suggesting that they are recently acquired, lineage-specific genes restricted to *Hepatoplasmataceae.* Lipases are thought to be essential for the survival of mollicutes as they are unable to synthesize fatty acids by themselves [81]. Phospholipases can also be virulence factors impacting host colonization and pathogenicity [90, 91]. Phospholipases are also a component of CBASS (cyclic oligonucleotide-based antiphage signalling system), a bacterial defence system against phages [92]. The functional importance of the patatin-like phospholipase in *Hepatoplasmataceae* warrant further investigation.

Overall, given the absence of polysaccharide-degrading enzymes in the *Hepatoplasmataceae* MAGs, it is unlikely that *Hepatoplasmataceae* benefit the host by aiding carbohydrate digestion, at least with proteins with detectable sequence similarities to known enzymes. This leaves the possibility that *Hepatoplasmataceae* might nutritionally benefit the host by means of other biomolecule-degrading enzymes such as nucleases, peptidases, and/or a lipase, although these enzymes might serve for the scavenging of nutrients for the bacteria’s own good.

### Genes possibly associated with host-bacterial interactions

We further explored for genes possibly associated with host-symbiont interactions. We used antiSMASH v. 6.1.1 [56] and NaPDoS2 server (https://npdomainseeker.sdsc.edu/napdos2/) [57] to screen for gene clusters related to secondary metabolite production, which might confer protection against certain pathogens. However, either search revealed no positive hits, suggesting that secondary metabolite production is not a defining characteristic of isopod-*Hepatoplasmataceae* symbiosis.

Endosymbiotic bacteria and some intracellular pathogens such as *Legionella* spp. are known to possess ELPs as a means of host-bacterial interactions [93, 94]. We searched for ELPs in *Hepatoplasmataceae* MAGs using EffectiveELD [66] and hmmscan [56]. The MAGs of *Candidatus* Hepatoplasma crinochetorum Tokyo2021 and *Candidatus* Hepatoplasma scabrum Ps-JP were found to encode seven and ten proteins, respectively, that bore a slight similarity to the regulator of chromosome condensation (RCC1) repeat (Table S5). RCC1 is a seven-bladed beta-propeller domain associated with various functions such as guanine nucleotide exchange on small GTP-binding proteins, enzyme inhibition or interaction with proteins and lipids [95]. A ColabFold 3D structure prediction for one of these homologs (HCTKY_2170; BDV02923.1; 991 amino acids; pLDDT=84; pTM=0.566; Fig. S1; BDV02923.1.zip) exhibited a seven-blade beta-propeller domain at its N-terminal, accompanied by four immunoglobulin (Ig)-like folds. Further analysis using FoldSeek to query the 3D model of BDV02923.1 showed similarities to beta-propeller proteins in both eukaryotes and prokaryotes, suggesting that the *Hepatoplasmataceae* beta-propeller proteins' resemblance to RCC1 repeats may be more about structural similarity at the fold level, rather than functional parallels or recent horizontal gene transfers from eukaryotic hosts. To summarize, although definitive proof of ELPs in *Hepatoplasmataceae* was not established, our search uncovered notable proteins that might be involved with host-symbiont interactions.

**Fig. 5.**
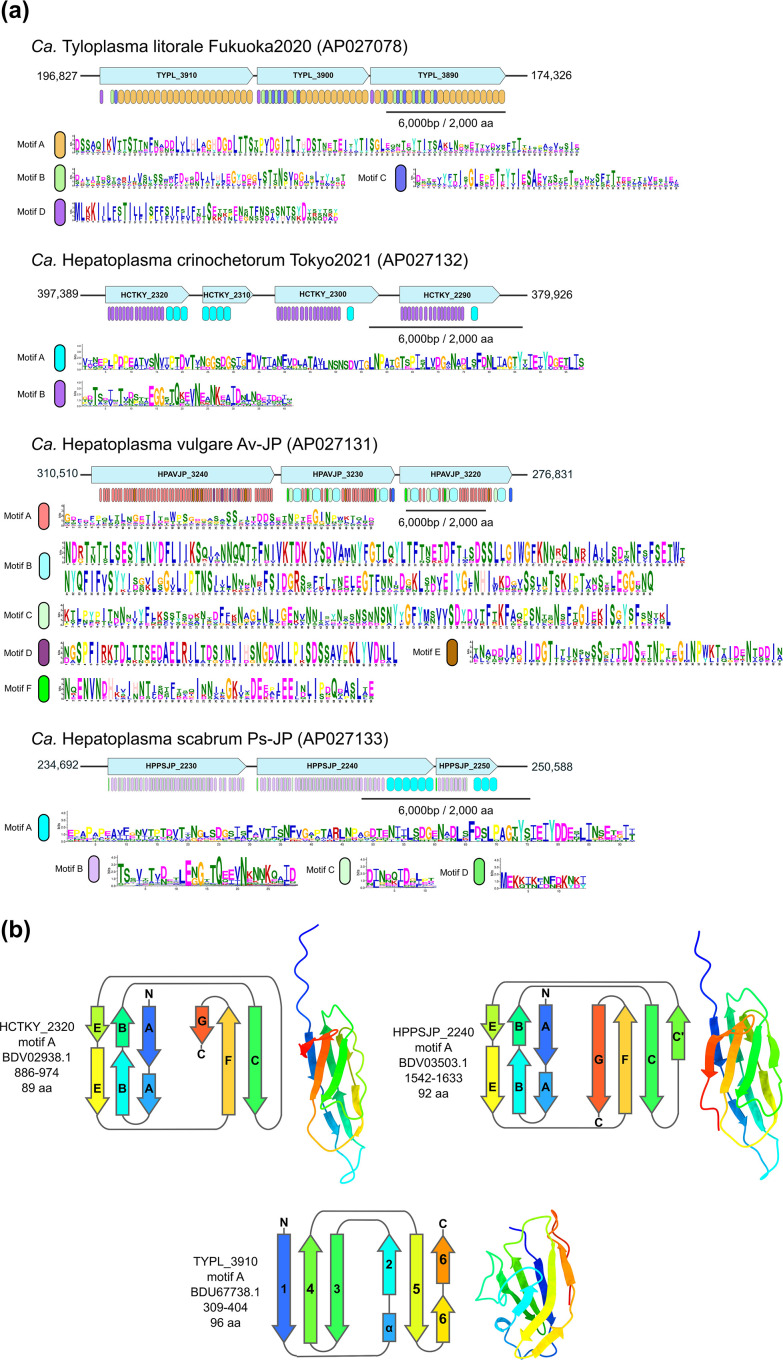
Large repetitive ORFs in *Hepatoplasmataceae* MAGs. (**a**) Diagrams of large, repetitive ORFs in *Hepatoplasmataceae* MAGs. Light blue arrows indicate the protein-coding genes and their transcriptional orientations. Numbers at the left and right ends indicate the start and end coordinates of the genome segments shown in the figure. Vertical rounded rectangles indicate the locations of repetitive motifs. (**b**) Schematic (left) and ColabFold 3D structure prediction (right) of beta-sandwich domains in the large repetitive ORFs of *Hepatoplasmataceae* MAGs. The sequences are coloured from blue at the N terminus to red at the C terminus. Arrows represent beta-strands, helices are shown as cylinders, and the loops are drawn as grey lines. Beta-strands forming Ig-like folds (HCTKY_2320 and HPPSJP_2440) are numbered alphabetically according to [111]. The numbering of beta-strands in TYPL_3910 is arbitrary.

Another striking feature of *Hepatoplasmataceae* MAGs is the presence of three to four large, repetitive ORFs (ORFs) organized in tandem clusters (Fig. 5). These proteins shared only limited sequence similarity with proteins outside the *Hepatoplasmataceae* family, bearing more resemblance to paralogs within their own genome. This pattern indicates that these gene clusters may have evolved independently within each species, although some cross-species amino acid level similarities were noted within *Candidatus* Hepatoplasma. The giant ORFs of *Candidatus* Tyloplasma litorale, in contrast, showed no sequence similarity to those in *Candidatus* Hepatoplasma spp., corroborating the idea of their distinct evolutionary origins. A MEME motif search identified series of tandem repeats ranging from 11 to 195 amino acids, with varying degrees of sequence conservation between unit types (Fig. 5a, b). The MEME domain classifications were manually refined to accurately represent beta-sandwich domains (discussed later) as separate entities (Fig. 5b). Most of these giant ORFs were too large to predict their 3D structures for whole sequences, but partial 3D structures for select repeat units were successfully predicted through alignments of these units (Fig. 5b, HCTKY_2320_Ig.zip, HPPSJP_2240_Ig.zip, TYPL_3910_sandwich.zip). These structural predictions revealed that segments of giant ORFs from *Candidatus* Hepatoplasma scaber and *Candidatus* Hepatoplasma crinochetorum contained tandem arrays of immunoglobulin (Ig)-like folds, a type of beta-sandwich domain, while the repeat units in *Candidatus* Tyloplasma litorale proteins exhibited a unique beta-sandwich structure. However, 3D structures for the repeat units in *Candidatus* Hepatoplasma vulgare ORFs could not be accurately predicted, even for individual units.

## Discussion

Isopod-associated mollicutes have attracted researchers’ attention due to the unique lifestyle of the host, which have successfully colonized land, and their possible contribution to the host’s success. Despite its unique standing in the known diversity of host-microbial interactions, our understanding on *Hepatoplasmataceae* has remained fragmentary. In this study, we sought to expand our knowledge on *Hepatoplasmataceae* by providing four complete *Hepatoplasmataceae* MAGs, three of which are potentially novel species, and pinpoint their possible roles in the isopod-mollicute symbiosis. While the exact tissue locations of *Hepatoplasmataceae* bacteria characterized in this study have not been microscopically determined, they are likely located on digestive organ surfaces, similar to other hepatoplasmas [15, 19]. The *Hepatoplasmataceae* MAGs assembled in this study are characterized by small genome sizes with highly streamlined genome architectures and lacked genes associated with major metabolic pathways.

Traditionally, isopod-mollicute symbiosis has been considered nutritional, with symbionts offering dietary benefits, such as aiding polysaccharide digestion or providing essential cofactors, to the host [12, 14, 43]. However, the *Hepatoplasmataceae* MAGs encode a limited variety of enzyme genes that could benefit the host nutritionally, notably lacking polysaccharide-degrading enzymes or secondary metabolite gene clusters. The only carbohydrate-degrading enzyme identified in this study’s *Hepatoplasmataceae* MAGs was an alpha,alpha-phosphotrehalase, a GH13 enzyme, in *Candidatus* Hepatoplasma spp. *Hepatoplasmataceae* MAGs Bg1 and Bg2 from deep-sea isopod *Bathynomus* sp. contain multiple copies of dipeptidases and GH1 and GH4 glucosidases, potentially aiding host survival in nutrient-scarce environments [21]. While these enzymes could benefit the isopod host, their limited presence in *Hepatoplasmataceae* suggests they are not essential for symbiosis. Overall, comparative genomic analysis challenges the view that carbohydrate digestion drives isopod-mollicute symbiosis, raising possibilities of other digestive contributions or non-nutritional symbiotic roles. It is also conceivable that hepatoplasmas encode unidentified enzymes, not detected by current homology search algorithms, an avenue for future research.

The assembly of the four MAGs utilized distinct methods, tailored to the size, complexity of the read data, and the prevalence of the bacterial reads in each sample. *Candidatus* Tyloplasma litorale Fukuoka2020 and *Candidatus* Hepatoplasma crinochetorum Toyko2021 MAGs were obtained as circular contigs from ONT assemblies. Polishing these assemblies with Illumina reads was relatively straightforward due to their dominance in the bacterial community of their respective datasets. However, for the *Candidatus* Hepatoplasma vulgare Av-JP MAG, also obtained as a circular contig from the ONT assembly, additional manual work was necessary. This involved using both an unpolished ONT assembly and an Illumina-based assembly to patch rRNA and tRNA genes. This correction was essential because these gene regions were highly similar to those in *Candidatus* Hepatoplasma crinochetorum Toyko2021, which had a much deeper sequencing coverage. This similarity led to the creation of chimeric sequences in the process of read mapping and during the consensus calling phase. The *Candidatus* Hepatoplasma scabrum Ps-JP MAG was successfully assembled into a circular contig using solely Illumina reads. While it is rather uncommon to derive complete genome sequences from Illumina-only assemblies, the uniformity in read coverage and the lack of indications of incompleteness or contamination in this assembly reinforce our belief in its completeness. In essence, the process of characterizing each MAG required different amounts of manual editing, depending on the nature of the dataset and the unique attributes of the genome being targeted.

Some symbiotic bacteria and intracellular pathogens encode ELPs that facilitate host-bacterial interactions. Sponge endosymbionts of *Poribacteria* phylum are known to be enriched with ELPs such as ankyrin, leucine-rich and tetratricopeptide repeat-containing proteins [93], many of which are involved in host-symbiont interactions. The genomes of intracellular pathogen *Legionella* spp. encode numerous ELPs that serve as virulence factors in human infection [94]. Although definitive ELPs were identified in the *Hepatoplasmataceae* MAGs, this is not unreasonable given that they are supposedly ectosymbionts.

The presence of multiple large repetitive proteins with predicted beta-sandwich structures is significant, because these structural domains are often found in adhesins, which play important roles in host cell adhesion [96–99]. Although we cannot categorically affirm their functions, it is possible that giant hypothetical proteins encoded by *Hepatoplasmataceae* MAGs play important roles in establishing isopod-mollicute symbiosis.

Symbiotic relationships are not always nutritional; some bacterial symbionts benefit the host by conferring resistance against invading pathogens, collectively called defensive symbionts [15, 100, 101]. Defensive symbionts often encode toxin genes to kill competing microorganisms [102], but *Hepatoplasmataceae* do not seem to encode toxins or any other secondary metabolite-producing gene clusters. This raises the possibility that they might adopt alternative defensive strategies. These could include forming physical barriers against competitor access to host tissues, monopolizing nutrients, or bolstering the host’s immune system through immune priming. An illustrative case is the *Spiroplasma* endosymbiont in *Drosophila*, which protects against parasitoid wasps by competing for lipids [84].

The digestive tracts of various metazoans including arthropods (insects and decapod crustaceans) and fishes [103–106] are lined by the peritrophic membrane, a chitinous layer that represent an important line of defence against pathogens. The loss of this chitin-based barrier in terrestrial vertebrates has been associated with the establishment of gut microbiota as an alternative defence mechanism [104]. Interestingly, the digestive organs of terrestrial isopods lack the peritrophic membrane [107, 108]. Wang *et al.* were first to note that *Candidatus* Hepatoplasma crinochetorum resides on the brush borders of hepatopancreas to line the surface [109]. In the deep-sea shrimp *Rimicaris* spp., *Hepatoplasmataceae* bacteria formed a thick mat on the surface of the foregut [110], a location where the peritrophic membrane is absent. The convergence of these findings in different *Hepatoplasmataceae* species, along with the lack of strong evidence for nutritional symbiosis from this study, suggests that *Hepatoplasmataceae* bacteria may function as a physical barrier. By colonizing the surfaces of the digestive tract, they likely play an important role in preventing pathogenic bacterial colonization. Therefore, *Hepatoplasmataceae* may be part of a defensive system that compensates for the absence of the peritrophic membrane, thereby aiding the host’s survival.

Overall, the availability of new data on isopod-associated mollicutes has provided valuable insights into the evolution of *Hepatoplasmataceae*. However, it is important to note that the analyses in this study are based on a limited number of genome sequences and lack experimental validation. Further sequencing and characterization of additional *Hepatoplasmataceae* lineages would greatly improve our understanding of the evolution and significance of the isopod-mollicute symbiosis.

### Description of Tyloplasma gen. nov.

Tyloplasma (Ty.lo.plas’ma. Gr comp. *Tylos*, referring to the host isopod *Tylos*; Gr. neut. n. *plasma*, something formed or moulded; N.L. neut. n. *tyloplasma*, intended to show association with the host isopod *Tylos*). The type species is *Candidatus* Tyloplasma litorale gen. nov. sp. nov. This genus corresponds to placeholder genus ‘g_Bg2’ in the release r214 of the Genome Taxonomy Database. The members of this genus can be distinguished from other species in the family *Hepatoplasmataceae* and order *Mycoplasmoidales* by their phylogenetic positions based on single-copy protein-coding genes and 16S rDNA sequences. Members of this genus are primarily found from digestive organs of semiterrestrial (e.g. *Tylos* spp. and *Ligia* spp.) and marine (*Bathynomus* sp.) isopods. Potential members of this genus have also been detected in marine metagenomic data.

### Description of ‘*Candidatus* Tyloplasma litorale’

Tyloplasma litorale (li.to.ra’le. L. neut. adj. *litorale,* referring to the littoral habitat of the host). This taxon is represented by the MAG AP027078.1 (CheckM2 v.1.0.1 completeness: 93.66 %; contamination: 0.09 %). This taxon is distinguished from other members of *Hepatoplasma* based on low average nucleotide identities, digital DNA–DNA hybridization values, and distinct phylogenetic positions. A likely ectosymbiont of the semiterrestrial isopod *Tylos granuliferus*. The circular genome is 615 622 bp in size with 24.4 % GC, coding for 530 protein-coding genes, six rRNA genes and 25 tRNA genes; lacks major metabolic pathways including biosynthesis of amino acids, nucleic acids, lipids, and cofactors; arginine deiminase pathway absent; predicted to utilize glucose, fructose, trehalose, *N*-Acetyl-d-glucosamine, and *N*-acetyl-muramic acid; so far uncultivated.

### Description of ‘*Candidatus* Hepatoplasma scabrum’

Hepatoplasma scabrum (sca'brum. L. neut. adj. *scabrum*, rough, scabrous; intended to show association with the host isopod *Porcellio scaber*). This taxon is represented by the MAG AP027133.1 (CheckM2 v.1.0.1 completeness: 96.31 %; contamination: 0.01 %). This taxon is distinguished from other members of *Hepatoplasma* based on low average nucleotide identities and digital DNA–DNA hybridization values. The circular genome is 606 194 bp in size with 24.7 % GC, coding for 543 protein-coding genes, three rRNA genes and 28 tRNA genes; lacks major metabolic pathways including biosynthesis of amino acids, nucleic acids, and cofactors; arginine deiminase pathway present; predicted to utilize glucose, fructose, trehalose, and *N*-acetyl-muramic acid, and glycerol; so far uncultivated.

### Description of ‘*Candidatus* Hepatoplasma vulgare’

Hepatoplasma vulgare (vul.ga're. L. neut. adj. *vulgare*, common;intended to show association with the host isopod *Armadillidium vulgare*). This taxon is represented by the MAG AP027131.1 (CheckM2 v.1.0.1 completeness: 90.16 %; contamination: 0.5 %). This taxon is distinguished from other members of *Hepatoplasma* based on low average nucleotide identities and digital DNA–DNA hybridization values. The circular genome is 662 079 bp in size with 22.7 % GC, coding for 597 protein-coding genes, three rRNA genes and 26 tRNA genes; lacks major metabolic pathways including biosynthesis of amino acids, nucleic acids, and cofactors; arginine deiminase pathway present; predicted to utilize glucose, fructose, trehalose, *N*-acetyl-muramic acid, and glycerol; so far uncultivated.

## Supplementary Data

Supplementary material 1
