# Supplementary material for: Metagenome-assembled genomes of three Hepatoplasmataceae provide insights into isopod-mollicute symbiosis
Source: Access Microbiol. 2024 Feb 20;6(2):000592.v3. doi: 10.1099/acmi.0.000592.v3 (PMC10928387; doi:10.1099/acmi.0.000592.v3)
Supplement: Supplementary material 1 [file acmi-6-592.v3-s001.pdf]

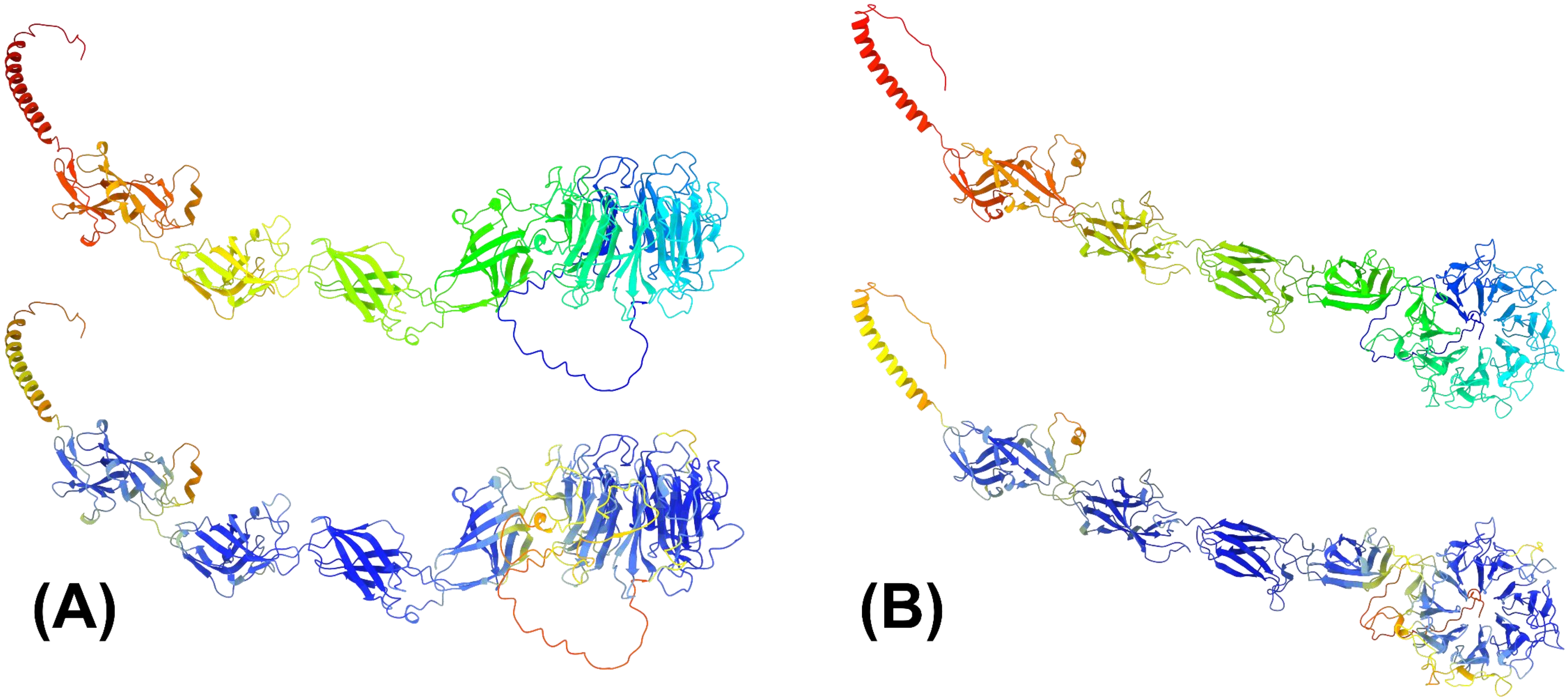

Supplementary Figure 1. ColabFold 3D prediction of beta-propeller-conteining protein HCTKY_2170

(A) Side view of HCTKY_2170's predicted 3D model with the upper image displaying a gradient from blue at the N-terminus to red at the C-terminus, denoting the linear sequence of the protein. The lower image is color-coded based on pLDDT confidence scores from ColabFold, with the spectrum ranging from blue (high confidence) to red (low confidence), representing the model's predicted reliability.

(B) Top view of the HCTKY_2170 model, highlighting the spatial configuration of its seven-blade beta-propeller structure.

Supplementary Table 1. Summary of PhyloFlash analysis results for quality-trimmed Illumina reads

		Armadillidium vulgare	***Porcellio scaber***	***Tylos granuliferus***
	SRA Accession	DRR394921	DRR394922	DRR394944
Mapping statistics	Input PE-reads	68,120,326	72,473,432	79,078,366
	Mapped SSU read pairs	60,242	43,946	27,943
	Mapping ratio	0.09%	0.06%	0.04%
Taxonomic affiliation of SSU rRNA reads in library	NTUs observed once	51	63	80
	NTUs observed twice	24	29	21
	NTUs observed three or more times	66	94	77
	NTU Chao1 richness estimate	120.188	162.431	229.381

Supplementary Table 2. Summary of read abundance and taxonomic assignments for 16S rDNA sequences assembled by PhyloFlash

	Entry	Length	Reads mapped	Mean depth	Mapped %	BLASTN top hit description	Accession	Identity%
A. vulgare	DRR394921.PFspades_1_499.389881	1,449	56,774	5643.6	47.12	*Armadillidium vulgare* 18S rRNA gene*	AJ287061.1	100.00%
	DRR394921.PFspades_2_81.254492	1,534	7,309	688.4	6.07	*Candidatus* Hepatoplasma crinochetorum Tokyo2021 DNA, complete genome*	AP027132.1	100.00%
	DRR394921.PFspades_3_13.393982	1,551	1,242	114.5	1.03	*Halomonas cupida* strain NBRC 102219 16S ribosomal RNA, partial sequence	NR_114046.1	96.83%
	DRR394921.PFspades_4_6.440056	1,556	568	52.1	0.47	*Rickettsiella massiliensis* 20B 16S ribosomal RNA, partial sequence	NR_117407.1	93.99%
	DRR394921.PFspades_5_7.173333	1,458	689	65.9	0.57	*Paracoccus methylarcula* strain h1 16S ribosomal RNA, complete sequence	NR_024866.2	97.53%
	DRR394921.PFspades_6_3.617203	1,519	291	27.2	0.24	*Candidatus* Hepatoplasma sp. Av-JP DNA, complete genome*	AP027131.1	99.93%
	DRR394921.PFspades_7_2.786506	1,516	276	24.4	0.23	*Joostella marina* strain En5 16S ribosomal RNA, partial sequence	NR_044346.1	94.05%
	DRR394921.PFspades_8_3.026530	1,517	269	24.3	0.22	*Algoriphagus resistens* strain NH1 16S ribosomal RNA, partial sequence	NR_156890.1	96.16%
				Total mapped %	55.96			
P.scaber	DRR394922.PFspades_1_364.577999	1,450	38,706	3816.19	44.04	*Porcellio scaber* 18S rRNA gene*	AJ287062.1	99.59%
	DRR394922.PFspades_2_34.308066	1,786	3,517	283.92	4	*Plagiorhynchus cylindraceus* 18S ribosomal RNA gene, complete sequence*	AF001839.1	99.60%
	DRR394922.PFspades_3_4.127436	1,527	380	35.23	0.43	*Pseudorhodoferax soli* strain TBEA3 16S ribosomal RNA, partial sequence	NR_044574.1	96.92%
	DRR394922.PFspades_4_2.706253	1,528	213	19.94	0.24	*Candidatus* Hepatoplasma sp. Ps-JP DNA, complete genome*	AP027133.1	100.00%
	DRR394922.PFspades_5_2.181575	1,516	182	16.41	0.21	*Algoriphagus resistens* strain NH1 16S ribosomal RNA, partial sequence	NR_156890.1	94.48%
	DRR394922.PFspades_6_3.845349	1,460	398	38.13	0.45	*Paracoccus methylarcula* strain h1 16S ribosomal RNA, complete sequence	NR_024866.2	96.23%
	DRR394922.PFspades_7_1.196660	1,530	270	23.81	0.31	*Demetria terragena* strain HK1 0089 16S ribosomal RNA, partial sequence	NR_026425.1	97.54%
	DRR394922.PFspades_8_2.311765	1,214	154	17.47	0.18	*Vibrio rumoiensis* strain S-1 16S ribosomal RNA, partial sequence	NR_024680.1	99.07%
				Total mapped %	49.86			
T.granuliferus	DRR394944.PFspades_1_175.891710	1,411	18,795	1902.2	33.63	*Tylos ponticus* small subunit ribosomal RNA gene, partial sequence*	GQ302707.1	98.48%
	DRR394944.PFspades_2_99.178834	1,524	9,013	855.4	16.13	*Mycoplasmatales* bacterium Fukuoka2020 DNA, complete genome*	AP027078.1	99.93%
	DRR394944.PFspades_3_4.287313	1,458	630	58.5	1.13	*Sedimentimonas flavescens* strain B57 16S ribosomal RNA, partial sequence	NR_181779.1	93.45%
	DRR394944.PFspades_4_7.415208	1,539	782	69.5	1.4	*Vibrio penaeicida* strain DSM 14398 16S ribosomal RNA, partial sequence	NR_042121.1	96.68%
	DRR394944.PFspades_5_1.739726	1,485	218	19.2	0.39	*Sphingopyxis witflariensis* strain W-50 16S ribosomal RNA, partial sequence	NR_028010.1	96.09%
	DRR394944.PFspades_6_1.930939	1,541	207	17.7	0.37	*Wenzhouxiangella salilacus* strain 15181 16S ribosomal RNA, partial sequence	NR_164943.1	90.77%
	DRR394944.PFspades_7_4.079928	1,539	458	40.9	0.82	*Granulosicoccus coccoides* strain Z 271 16S ribosomal RNA, partial sequence	NR_104509.1	90.95%
	DRR394944.PFspades_8_1.197568	1,506	465	34.6	0.83	*Zobellia galactanivorans* strain DsiJ 16S ribosomal RNA, partial sequence	NR_074684.1	86.79%
	DRR394944.PFspades_9_2.010652	1,485	206	17.9	0.37	*Minwuia thermotolerans* strain SY3-15 16S ribosomal RNA, partial sequence	NR_179861.1	90.55%
	DRR394944.PFspades_10_0.936262	1,286	116	11.9	0.21	*Aequoribacter fuscus* strain IMCC3088 16S ribosomal RNA, partial sequence	NR_173548.1	94.25%
				Total mapped %	55.07			

BLASTN top hits represent the primary matches from a BLASTN search against the NCBI 16S rRNA sequences database (Bacteria and Archaea). Entries with an asterisk (*) denote hits derived from the nonredundant nucleotide database.

Supplementary Table 3. GTDB-tk analysis results for *Hepatoplasmataceae* MAGs

Species	*Ca.* Tyloplasma litorale	*Ca.* Hepatoplasma vulgare	*Ca.* Hepatoplasma scabrum	*Ca.* Hepatoplasma crinochetorum
Isolate	Fukuoka2020	Av-JP	Ps-JP	Tokyo2021
user_genome	AP027078.1	AP027131.1	AP027132.1	AP027133.1
classification	d__Bacteria; p__Bacillota; c__Bacilli; o__Mycoplasmatales; f__Hepatoplasmataceae; g__Bg2; s__	d__Bacteria; p__Bacillota; c__Bacilli; o__Mycoplasmatales; f__Hepatoplasmataceae; g__Hepatoplasma; s__	d__Bacteria; p__Bacillota; c__Bacilli; o__Mycoplasmatales; f__Hepatoplasmataceae; g__Hepatoplasma; s__Hepatoplasma crinochetorum_B	d__Bacteria; p__Bacillota; c__Bacilli; o__Mycoplasmatales; f__Hepatoplasmataceae; g__Hepatoplasma; s__
fastani_reference	N/A	N/A	GCF_000582535.1	N/A
fastani_reference_radius	N/A	N/A	95	N/A
fastani_taxonomy	N/A	N/A	d__Bacteria; p__Bacillota; c__Bacilli; o__Mycoplasmatales; f__Hepatoplasmataceae; g__Hepatoplasma; s__Hepatoplasma crinochetorum_B	N/A
fastani_ani	N/A	N/A	98.43	N/A
fastani_af	N/A	N/A	0.98	N/A
closest_placement_reference	GCA_013214765.1	N/A	GCF_000582535.1	GCA_001179805.1
closest_placement_radius	95	N/A	95	95
closest_placement_taxonomy	d__Bacteria; p__Bacillota; c__Bacilli; o__Mycoplasmatales; f__Hepatoplasmataceae; g__Bg2; s__Bg2 sp013214765	N/A	d__Bacteria; p__Bacillota; c__Bacilli; o__Mycoplasmatales; f__Hepatoplasmataceae; g__Hepatoplasma; s__Hepatoplasma crinochetorum_B	d__Bacteria; p__Bacillota; c__Bacilli; o__Mycoplasmatales; f__Hepatoplasmataceae; g__Hepatoplasma; s__Hepatoplasma crinochetorum_A
closest_placement_ani	78.7	N/A	98.43	83.12
closest_placement_af	0.39	N/A	0.98	0.85
pplacer_taxonomy	d__Bacteria; p__Bacillota; c__Bacilli; o__Mycoplasmatales; f__Hepatoplasmataceae; g__Bg2; s__	d__Bacteria; p__Bacillota; c__Bacilli; o__Mycoplasmatales; f__Hepatoplasmataceae; g__Hepatoplasma; s__	d__Bacteria; p__Bacillota; c__Bacilli; o__Mycoplasmatales; f__Hepatoplasmataceae; g__Hepatoplasma; s__	d__Bacteria; p__Bacillota; c__Bacilli; o__Mycoplasmatales; f__Hepatoplasmataceae; g__Hepatoplasma; s__
classification_method	taxonomic classification defined by topology and ANI	taxonomic novelty determined using RED	taxonomic classification defined by topology and ANI	taxonomic classification defined by topology and ANI
note	classification based on placement in class-level tree	classification based on placement in class-level tree	topological placement and ANI have congruent species assignments	classification based on placement in class-level tree
other_related_references (genome_id, species_name, radius, ANI, AF)	GCA_013139135.1, s__Bg2 sp013139135, 95.0, 77.13, 0.11; GCA_001641225.1, s__Bg2 sp001641225, 95.0, 76.62, 0.17; GCA_001641205.1, s__Bg2 sp001641205, 95.0, 75.94, 0.18	N/A	GCA_001179805.1, s__Hepatoplasma crinochetorum_A, 95.0, 82.67, 0.83	GCF_000582535.1, s__Hepatoplasma crinochetorum_B, 95.0, 81.29, 0.82
msa_percent	74.66	73.53	74.44	75.45
translation_table	4	4	4	4
red_value	0.942229691	0.89349363	N/A	0.978868018
warnings	N/A	N/A	N/A	Genome not assigned to closest species as it falls outside its pre-defined ANI radius

Supplementary Table 4. Nucleases, peptidases, and a lipase found in *Hepatoplasmataceae* MAGs

			Fukuoka2020	Av-JP	Ps-JP	Tokyo2021
Nuclease	DNA maintenance	DNA polymerase I	BDU67453.1<br>BDU67375.1	BDV02568.1	BDV03711.1	BDV03161.1
		DNA polymerase III PolC-type	BDU67697.1	BDV02592.1	BDV03442.1	BDV02873.1
		recombination protein U	BDU67478.1	BDV02296.1	BDV03369.1	BDV02798.1
		deoxyribonuclease IV	BDU67583.1	BDV02352.1<br>BDV02356.1	BDV03466.1	BDV02896.1
		DNA-binding protein WhiA	BDU67830.1	BDV02655.1	BDV03677.1	BDV03127.1
		UvrABC system protein A	BDU67671.1	BDV02541.1	BDV03748.1	BDV03199.1
		UvrABC system protein B	BDU67672.1	BDV02547.1	BDV03751.1	BDV03202.1
		UvrABC system protein C	BDU67670.1	BDV02533.1	BDV03426.1	BDV02857.1
		hydrolase TatD	BDU67849.1	BDV03263.1	BDV03806.1	BDV02118.1
	Rnase	ribonuclease J	BDU67468.1<br>BDU67637.1	BDV02285.1<br>BDV02558.1	BDV03359.1<br>BDV03511.1	BDV02787.1<br>BDV02960.1
		ribonuclease R	BDU67412.1	BDV02138.1	BDV03304.1	BDV02732.1<br>BDV02733.1
		Ribonuclease III	BDU67510.1	BDV02239.1	BDV03718.1	BDV03166.1
		Endoribonuclease YbeY	BDU67801.1	BDV02335.1	BDV03586.1	BDV03034.1
		ribonuclease HIII	BDU67688.1	BDV02255.1		
		ribonuclease P	BDU67876.1	BDV02706.1	BDV03821.1	BDV03278.1
		pre-16S rRNA nuclease	BDU67626.1	BDV02449.1	BDV03554.1	BDV03002.1
	CRISPR/Cas9	Cas1	BDU67747.1	BDV02176.1	BDV03524.1	BDV02971.1
		Cas2 2	BDU67748.1	BDV02174.1	BDV03523.1	BDV02970.1
		Cas9	BDU67745.1	BDV02189.1	BDV03530.1	BDV02979.1
		Csn2			BDV03798.1	BDV03798.1
	Genome defence	type I restriction endonuclease subunit R	BDU67870.1	BDV02406.1		BDV02944.1
		Type I restriction-modification system subunit M	BDU67867.1			BDV02945.1
		RecB family nuclease	BDU67612.1	BDV02427.1	BDV03668.1	BDV03118.1
		type II restriction endonuclease SauIIIAI		BDV02278.1		
		type II restriction endonuclease MjaI	BDU67724.1			
		type II restriction endonuclease DpnII	BDU67733.1			
	Unknown	nuclease/helicase DNA2			BDV03810.1	BDV03267.1
		DNA helicase TIGR00376			BDV03803.1	
		exonuclease V	BDU67746.1			
		S-adenosylmethionine-dependent methyltransferase		BDV02401.1		
		Zn-dependent hydrolase/glyoxylase		BDV02540.1	BDV03628.1	BDV03075.1
		GIY-YIG type nuclease	BDU67517.1<br>BDU67518.1		BDV03716.1<br>BDV03613.1	BDV02805.1<br>BDV02839.1<br>BDV03061.1
		HNH nucleases		BDV02640.1<br>BDV02539.1	BDV03587.1<br>BDV03771.1<br>BDV03775.1	BDV03035.1<br>BDV03182.1
Peptidase	Housekeeping genes	ATP-dependent metalloprotease FtsH	BDU67403.1	BDV02127.1	BDV03292.1	BDV02721.1
		ATP-dependent serine endopeptidase La	BDU67465.1	BDV02282.1	BDV03356.1	BDV02784.1
		ribosomal-processing cysteine protease Prp	BDU67622.1	BDV02455.1	BDV03549.1	BDV02997.1
		RIP metalloprotease RseP		BDV02593.1	BDV03441.1	BDV02872.1
	Unknown	M18 peptidase aminopeptidase family	BDU67643.1<br>BDU67549.1	BDV02274.1<br>BDV02578.1	BDV03345.1<br>BDV03514.1	BDV02963.1<br>BDV02773.1
		Putative neutral zinc metallopeptidase	BDU67476.1	BDV02293.1	BDV03367.1	BDV02796.1
		Xaa-Pro aminopeptidase	BDU67599.1	BDV02383.1	BDV03701.1	BDV03151.1
		methionine aminopeptidase, type I	BDU67446.1	BDV02617.1	BDV03573.1	BDV03021.1
		M20 Peptidase Xaa-His dipeptidase (PepV)	BDU67609.1<br>BDU67717.1	BDV02610.1		
Lipase		Patatin-like phospholipase	BDU67589.1	BDV02373.1	BDV03688.1	BDV03138.1

Supplementary Table 5. Beta-barrel-containing proteins in *Ca.* Hepatoplasma MAGs

Species	Accession no.	ID
Ca. Hepatoplasma crinochetorum	BDV02923.1	HCTKY_2170
	BDV02924.1	HCTKY_2180
	BDV02939.1	HCTKY_2330
	BDV02951.1	HCTKY_2450
	BDV02981.1	HCTKY_2750
	BDV02982.1	HCTKY_2760
	BDV03256.1	HCTKY_5500
Ca. Hepatoplasma scabrum Ps-JP	BDV03469.1	HPPSJP_1900
	BDV03478.1	HPPSJP_1990
	BDV03497.1	HPPSJP_2180
	BDV03501.1	HPPSJP_2220
	BDV03515.1	HPPSJP_2360
	BDV03516.1	HPPSJP_2370
	BDV03517.1	HPSSJP_2380
	BDV03533.1	HPPSJP_2540
	BDV03534.1	HPPSJP_2550
	BDV03773.1	HPPSJP_4940
